# Association of missing paternal demographics on infant birth certificates with perinatal risk factors for childhood obesity

**DOI:** 10.1186/s12889-016-3110-1

**Published:** 2016-07-14

**Authors:** Erika R. Cheng, Summer Sherburne Hawkins, Sheryl L. Rifas-Shiman, Matthew W. Gillman, Elsie M. Taveras

**Affiliations:** Department of Pediatrics, Children’s Health Services Research, Indiana University School of Medicine, 410 West 10th Street, Suite 2000, Indianapolis, IN USA; Boston College, School of Social Work, McGuinn Hall, 140 Commonwealth Avenue, Chestnut, Hill, MA USA; Department of Population Medicine, Obesity Prevention Program, Harvard Medical School and Harvard Pilgrim Health Care Institute, 401 Park Drive, Suite 401, Boston, MA USA; Department of Pediatrics, Division of General Academic Pediatrics, Massachusetts General Hospital for Children, 125 Nashua Street, Suite 860, Boston, MA USA

**Keywords:** Birth certificates, Electronic health records, Health status disparities, Medical record linkage, Pediatric obesity, Paternal factors, Perinatal health

## Abstract

**Background:**

The role of fathers in the development of obesity in their offspring remains poorly understood. We evaluated associations of missing paternal demographic information on birth certificates with perinatal risk factors for childhood obesity.

**Methods:**

Data were from the Linked CENTURY Study, a database linking birth certificate and well-child visit data for 200,258 Massachusetts children from 1980–2008. We categorized participants based on the availability of paternal age, education, or race/ethnicity and maternal marital status on the birth certificate: (1) pregnancies missing paternal data; (2) pregnancies involving unmarried women with paternal data; and (3) pregnancies involving married women with paternal data. Using linear and logistic regression, we compared differences in smoking during pregnancy, gestational diabetes, birthweight, breastfeeding initiation, and ever recording a weight for length (WFL) ≥ the 95th percentile or crossing upwards ≥2 WFL percentiles between 0–24 months among the study groups.

**Results:**

11,989 (6.0 %) birth certificates were missing paternal data; 31,323 (15.6 %) mothers were unmarried. In adjusted analyses, missing paternal data was associated with lower birthweight (β -0.07 kg; 95 % CI: −0.08, −0.05), smoking during pregnancy (AOR 4.40; 95 % CI: 3.97, 4.87), non-initiation of breastfeeding (AOR 0.39; 95 % CI: 0.36, 0.42), and with ever having a WFL ≥ 95th percentile (AOR 1.10; 95 % CI: 1.01, 1.20). Similar associations were noted for pregnancies involving unmarried women with paternal data, but differences were less pronounced.

**Conclusions:**

Missing paternal data on the birth certificate is associated with perinatal risk factors for childhood obesity. Efforts to understand and reduce obesity risk factors in early life may need to consider paternal factors.

## Background

The prevention of obesity is a national and global health priority [1, 2]. In the United States, 16.9 % of children ages 2–19 years and more than one-third of adults are obese (body mass index [BMI] ≥ 95th percentile) [3]. In children, early obesity and excess weight gain not only predict later obesity and cardio-metabolic risk, but also serious childhood morbidities, including asthma, orthopedic problems, psychosocial adversity, and increasingly, Type 2 diabetes [4–6]. Epidemiologic studies suggest that adverse exposures in the intrauterine and infancy periods can “program” trajectories of adiposity and metabolic function throughout life [7, 8] and may increase short- and long-term risks for obesity and its sequelae. Pregnancy and early childhood are thus critical periods for obesity prevention, and identifying early risk factors is essential to inform intervention efforts.

Many perinatal risk factors implicated in childhood obesity lie within the family context [9]. Maternal pre-pregnancy weight, gestational weight gain, smoking during pregnancy, and depression have all been associated with obesity in offspring [10]. Although less well studied, paternal factors may also influence children’s obesity [11]. For example, father engagement in prenatal activities and reports of providing emotional and financial support are associated with a lower likelihood of low birthweight [12–14], an important precursor of children’s metabolic risk. Women whose partners are involved in their pregnancies are also more likely to receive early prenatal care and reduce cigarette smoking [15, 16]. Further, a recent review of child feeding research found that fathers are less likely than mothers to monitor children’s food intake and to limit access to food [17], suggesting that fathers contribute directly to obesity risk factors within families in ways that are unique from mothers [17]. Studies assessing the effects of family structure on childhood obesity also support the potential importance of father involvement, for example linking childhood obesity with parental separation before birth [18] and with single-parent household status [19]. Despite this evidence, the literature on fathers’ contributions to children’s obesity outcomes is limited.

One valuable source of paternal data is birth certificates. Researchers have reported associations of missing paternal demographic information on the birth certificate, often conceptualized as a proxy for limited paternal involvement, with inadequate prenatal care [20], pregnancy complications [21], adverse birth outcomes [20, 22], and infant mortality [14, 23]. The purpose of this study was to extend this knowledge base to the field of childhood obesity. Specifically, we capitalized on the availability of a new, longitudinally-linked database of children living in Massachusetts to determine the extent to which missing paternal demographic information on birth certificates is associated with perinatal risk factors for childhood obesity, including maternal smoking during pregnancy, gestational diabetes, infant birthweight, breastfeeding initiation, and ever having a weight for length (WFL) ≥ 95th percentile or crossing upwards ≥2 WFL percentiles between 0–24 months of age.

## Methods

### Data source

We used data from the Linked CENTURY Study, a longitudinal electronic health record database linking birth certificate and well-child visit data for 200,343 Massachusetts children ages 0 to <18 years. Details of the Linked CENTURY Study are available elsewhere [24]. Briefly, the Linked CENTURY Study is the linkage of the existing CENTURY Study, a clinical database [25], with each child’s Massachusetts birth certificate. The Collecting Electronic Nutrition Trajectory Data Using e-Records of Youth (CENTURY) Study is a clinical database of 269,959 singleton children ages 0 to <18 years who were seen for a well-child visit at any of the 14 health centers of Atrius Health, including Harvard Vanguard Medical Associates (HVMA) and other smaller health centers in eastern Massachusetts from 1980 through 2008. All CENTURY participants were born between 1969 through 2008. The definition of a well-child visit was the use of an appropriate utilization code, the combination of measurement of weight and length or height, or administration of a routine immunization. The CENTURY study contains children’s demographic and clinical growth data (e.g., measured heights and weights), but lacks comprehensive data to assess maternal pregnancy history and detailed socio-demographic information of both mothers and fathers. Therefore, the study team created the Linked CENTURY Study by linking data from the existing CENTURY Study to each child’s Massachusetts birth certificate using an algorithm that compared the child’s name and date of birth and the mother’s date of birth. Overall, 74.2 % of the CENTURY cohort was matched, resulting in 200,343 children in the Linked CENTURY Study. Among this cohort, 60.9 % (121,917) of children had at least one other sibling in the dataset. We removed 85 cases missing birth certificate data on marital status, yielding a sample of 200,258.

Institutional Review Board approval for the CENTURY Study was obtained from Boston College, Harvard Pilgrim Health Care (HPHC), MDPH, and the Massachusetts General Hospital for Children.

### Measures

To create our main exposure variable, we categorized our study population based on the availability of paternal age, education, or race/ethnicity and the woman’s marital status on the birth certificate as: (1) pregnancies with missing paternal data; (2) pregnancies of unmarried women with paternal data available; and (3) pregnancies of married women with paternal data available. Accounting for marital status allowed us to independently assess the impact of paternal factors on our dependent measures. There were relatively few birth certificates (*N* = 368 or 0.2 %) missing paternal data where the mothers also reported being married, so we collapsed the groups with missing paternal data to create 3 exposure categories. Removing these cases from the sample had no appreciable impact on our findings (data not shown).

### Perinatal risk factors

Our main outcomes of interest, derived from the birth certificate, were: (1) maternal smoking during pregnancy (yes versus no based on the reported number of cigarettes per day, data available from 1992 through 2008); (2) whether the mother had gestational diabetes (data available from 1996 through 2008); (3) birthweight; and (4) whether the mother was breastfeeding at the time the birth certificate was completed (data available from 1996 to 2008; hereafter “breastfeeding initiation”). We also examined the prevalence and change in children’s age- and sex-specific weight for length (WFL) z-scores in the first 24 months of life using clinically measured length and weight during the well-child visit. We specifically examined: (1) whether the child ever recorded a WFL at or above the 95th percentile; and (2) whether the child crossed ≥2 major WFL percentiles in any of the following 4 six-month periods in the first 24 months of life: 1–6 months, 6–12 months, 12–18 months, and 18–24 months. This measure that has been associated with later obesity in the CENTURY cohort [26].

Covariates from the birth certificate included child sex and gestational age at birth. We assessed several maternal characteristics that are associated with our outcomes of interest, including: age (<35 years versus ≥35 years); race/ethnicity (white, black, Hispanic, and other); parity (nulliparous versus multiparous); education (less than high school, high school degree, some college, or college degree and higher); and insurance status (private versus other).

### Analytic approach

We first obtained means and percentages to describe the sample characteristics. We then used unadjusted and multivariable adjusted linear and logistic regression to examine the likelihood of maternal smoking during pregnancy, gestational diabetes, infant birthweight, breastfeeding initiation, and having a WFL at or above the 95th percentile or crossing upwards of ≥2 WFL percentiles between ages 0–24 months for two groups of pregnancies: (1) those with missing paternal data; and (2) those with paternal data where the mother reported being unmarried. Pregnancies of married mothers with paternal data available comprised the reference group. The first model estimated the unadjusted associations. The second model adjusted for child’s sex and gestational age at birth; model 3 additionally adjusted for maternal age, parity, and race/ethnicity; model 4 additionally adjusted for smoking during pregnancy; model 5 additionally adjusted for maternal education, and model 6 added maternal insurance status. We assumed that any diagnosis of gestational diabetes occurred prior to delivery, so we did not adjust for gestational age at birth in our multivariable model of gestational diabetes. Our models of maternal smoking during pregnancy did not adjust for gestational age at birth. As a sensitivity analysis, we then restricted the sample to a more recent time period (2004 to 2008); this modification did not influence the interpretation of our results (Appendix) so we present findings from the full sample. We performed data analyses with SAS v9.3 (SAS Institute, Cary, NC).

## Results

### Sample characteristics

Overall, 6.0 % (*N* = 11,989) of birth certificates were missing paternal data and 15.6 % (*N* = 31,323) of mothers were unmarried. The vast majority of pregnancies with available paternal data involved married mothers (89.6 %); 10.4 % (*N* = 19,611) of pregnancies with paternal data involved unmarried mothers.

Table 1 describes the distribution of child and maternal characteristics within each of the three study groups. Pregnancies with missing paternal data were most likely to involve women who were younger (92.8 % were <35 years old), of non-Hispanic black race/ethnicity (51.4 %), and who did not initiate breastfeeding (46.3 %).

**Table 1:** Sample Characteristics of Participants in the Linked CENTURY Study, Overall and by Exposure Categories

	Total *N* = 200,258	No paternal data^a^ *N* = 11,989	Paternal data, not married *N* = 19,611	Paternal data, married *N* = 168,658
	*N* (%)			
Maternal Characteristics				
Age (*n* = 199,909)				
<35 years	159250 (79.7)	11098 (92.8)	17787 (90.8)	130365 (77.4)
≥35 years	40659 (20.3)	855 (7.2)	1795 (9.2)	38009 (22.6)
Race/ethnicity (*n* = 197,710)				
White	9122 (4.6)	4086 (35.1)	10927 (56.2)	136616 (82.0)
Black	151629 (76.7)	5978 (51.4)	5154 (26.5)	12010 (7.2)
Hispanic	23142 (11.7)	1099 (9.4)	2405 (12.4)	5618 (3.4)
Other	13817 (7.0)	472 (4.1)	955 (4.9)	12390 (7.4)
Nulliparous (*n* = 181,569)				
No	96093 (52.9)	4168 (40.8)	7310 (39.8)	84615 (55.3)
Yes	85476 (47.1)	6038 (59.2)	11045 (60.2)	68393 (44.7)
Education (*n* = 155,096)				
Less than high school	2433 (1.2)	463 (4.0)	440 (2.3)	1530 (0.9)
High school degree	67139 (34.0)	7643 (66.8)	11299 (58.4)	48197 (28.9)
Some college	100245 (50.8)	2984 (26.1)	7074 (36.6)	90187 (54.1)
College degree and higher	27671 (14.0)	351 (3.1)	529 (2.7)	26791 (16.1)
Private Insurance (*n* = 197,488)				
No	19076 (12.3)	3950 (46.9)	7580 (44.0)	7546 (5.8)
Yes	136020 (87.7)	4478 (53.1)	9636 (56.0)	121906 (94.2)
Smoked during pregnancy^b^ (*n* = 116,697)				
No	109846 (94.1)	4071 (83.8)	11753 (82.6)	94022 (96.3)
Yes	6851 (5.9)	785 (16.2)	2469 (17.4)	3597 (3.7)
Gestational diabetes^c^ (*n* = 83,836)				
No	80952 (96.6)	2655 (96.9)	10711 (96.9)	67586 (96.5)
Yes	2884 (3.4)	85 (3.1)	346 (3.1)	2453 (3.5)
Initiated breastfeeding^c^ (*n* = 154,734)				
No	36096 (23.3)	3889 (46.3)	6377 (37.1)	25830 (20.0)
Yes	118638 (76.7)	4513 (53.7)	10795 (62.9)	103330 (80.0)
Child Characteristics				
Sex (*n* = 200,258)				
Male	100753 (50.3)	5946 (49.6)	9708 (49.5)	85099 (50.5)
Female	99505 (49.7)	6043 (50.4)	9903 (50.5)	83559 (49.5)
	Mean (SD)			
Birthweight, kg (*n* = 199,925)	3.4 (0.5)	3.2 (0.6)	3.3 (0.6)	3.5 (0.5)
Gestational age at birth, weeks (*n* = 179,139)	39.6 (2.1)	39.4 (2.7)	39.5 (2.4)	39.7 (2.0)
WFL ever ≥95th percentile 0–24 months^d^ (*n* = 104,540)				
Male	100753 (50.3)	5946 (49.6)	9708 (49.5)	85099 (50.5)
Female	99505 (49.7)	6043 (50.4)	9903 (50.5)	83559 (49.5)
Crossed ≥2 major WFL percentiles 0–24 months^e^ (*n* = 25,409)				
No	9147 (36.0)	233 (32.0)	616 (35.3)	8298 (36.2)
Yes	16262 (64.0)	494 (68.0)	1130 (64.7)	14638 (63.8)

*Notes*: *kg* kilograms, *WFL* weight-for-length, *SD* standard deviation

^a^Paternal birth certificate included age, race/ethnicity, or education status; 368 (3.1 %) of cases with missing paternal data on the birth certificate were married; ^b^On birth certificate 1992 to 2008; ^c^On birth certificate 1996 to 2008; ^d^Limited to participants with at least 1 recorded height/weight measurement between ages 0 to 24 months. ^e^Limited to participants with all 4 crossings between ages 0 to 24 months (e.g., 0–6, 6–12, 12–18, 18–24)

### Multivariable results

In unadjusted analyses (Table 2), the rate of smoking during pregnancy was 5.0 times higher (odds ratio [OR] 5.04; 95 % confidence interval [CI]: 4.64, 5.48) and the rate of breastfeeding initiation was 71 % lower (OR 0.29; 95 % CI: 0.28, 0.30) in pregnancies with missing paternal data than in pregnancies where paternal data were available. Infant birthweights were also lower among pregnancies with missing paternal data (β -0.23 kg; 95 % CI −0.24, −0.22). Children with missing paternal data were also more likely to ever have a WFL ≥ 95th percentile (OR 1.27; 95 % CI: 1.20, 1.35), and to cross ≥2 major WFL percentiles within the first 4 months of life (OR 1.20; 95 % CI: 1.03, 1.41). These patterns were also observed among pregnancies with available paternal data involving unmarried mothers, although the differences were less pronounced than the group missing paternal data. We did not observe group differences in the rate of maternal gestational diabetes.

**Table 2:** Unadjusted and Multivariable Adjusted Associations* of 3-category Exposure with Perinatal Risk Factors for Childhood Obesity

		Model 1	Model 2	Model 3	Model 4	Model 5	Model 6
Outcome	Exposure	OR (95 % CI)					
Smoked during pregnancy^a^							
	Missing paternal data	5.04 (4.64, 5.48)	5.04 (4.64, 5.48)	9.27 (8.45,10.17)	-	6.10 (5.55, 6.72)	4.40 (3.97, 4.87)
	Paternal data, not married	5.49 (5.20, 5.80)	5.49 (5.20, 5.80)	7.63 (7.19, 8.10)	-	5.43 (5.10, 5.77)	4.02 (3.75, 4.30)
	Paternal data, married	1.00 (Reference)	1.00 (Reference)	1.00 (Reference)	-	1.00 (Reference)	1.00 (Reference)
Gestational diabetes^b^							
	Missing paternal data	0.88 (0.71, 1.10)	0.88 (0.71, 1.10)	0.78 (0.62, 0.98)	0.74 (0.59, 0.93)	0.76 (0.61, 0.96)	0.79 (0.63, 1.00)
	Paternal data, not married	0.89 (0.79, 1.00)	0.89 (0.79, 1.00)	0.86 (0.76, 0.97)	0.82 (0.72, 0.93)	0.83 (0.73, 0.94)	0.86 (0.75, 0.98)
	Paternal data, married	1.00 (Reference)	1.00 (Reference)	1.00 (Reference)	1.00 (Reference)	1.00 (Reference)	1.00 (Reference)
Initiated breastfeeding^c^							
	Missing paternal data	0.29 (0.28, 0.30)	0.30 (0.29, 0.32)	0.23 (0.22, 0.24)	0.29 (0.27, 0.31)	0.36 (0.34, 0.39)	0.39 (0.36, 0.42)
	Paternal data, not married	0.42 (0.41, 0.44)	0.42 (0.40, 0.43)	0.36 (0.34, 0.37)	0.40 (0.38, 0.42)	0.48 (0.46, 0.50)	0.51 (0.48, 0.53)
	Paternal data, married	1.00 (Reference)	1.00 (Reference)	1.00 (Reference)	1.00 (Reference)	1.00 (Reference)	1.00 (Reference)
WFL ever ≥95th percentile, 0–24 mo^d^							
	Missing paternal data	1.27 (1.20, 1.35)	1.31 (1.23, 1.40)	1.13 (1.06, 1.21)	1.08 (0.99, 1.18)	1.09 (1.00, 1.18)	1.10 (1.01, 1.20)
	Paternal data, not married	1.17 (1.12, 1.23)	1.20 (1.15, 1.26)	1.10 (1.05, 1.16)	1.08 (1.02, 1.14)	1.08 (1.02, 1.14)	1.10 (1.04, 1.16)
	Paternal data, married	1.00 (Reference)	1.00 (Reference)	1.00 (Reference)	1.00 (Reference)	1.00 (Reference)	1.00 (Reference)
Crossed ≥2 major WFL percentiles, 0–24 mo^e^							
	Missing paternal data	1.20 (1.03, 1.41)	1.19 (1.01, 1.41)	1.16 (0.98, 1.38)	1.10 (0.91, 1.35)	1.11 (0.91, 1.35)	1.14 (0.93, 1.39)
	Paternal data, not married	1.04 (0.94, 1.15)	1.03 (0.93, 1.15)	1.02 (0.91, 1.13)	1.06 (0.94, 1.19)	1.06 (0.94, 1.19)	1.08 (0.96, 1.22)
	Paternal data, married	1.00 (Reference)	1.00 (Reference)	1.00 (Reference)	1.00 (Reference)	1.00 (Reference)	1.00 (Reference)
		β (95 % CI)					
Birthweight, kg							
	Missing paternal data	−0.23 (−0.24,-0.22)	−0.20 (−0.21,-0.19)	−0.13 (−0.14,-0.11)	−0.09 (−0.10,-0.08)	−0.08 (−0.10,-0.07)	−0.07 (−0.08,-0.05)
	Paternal data, not married	−0.14 (−0.15,-0.13)	−0.12 (−0.13,-0.11)	−0.07 (−0.08,-0.06)	−0.05 (−0.06,-0.04)	−0.05 (−0.06,-0.04)	−0.03 (−0.04,-0.02)
	Paternal data, married	0.00 (Reference)	0.00 (Reference)	0.00 (Reference)	0.00 (Reference)	0.00 (Reference)	0.00 (Reference)

*WFL weight-for-length, *kg*, kilograms *OR* odds ratio *CI* confidence interval. Model 1 is unadjusted. Model 2 adjusted for child gestational age at birth and sex. Model 3 added maternal age (≥35 years), parity (nulliparous), and race/ethnicity. Model 4 added smoking during pregnancy. Model 5 added maternal education. Model 6 added insurance status. Smoking during pregnancy and gestational diabetes models were not adjusted for gestational age at birth

^a^Smoking during pregnancy was on birth certificate 1992 to 2008; N decreases from Model 3 to Model 4.; ^b^On birth certificate 1987 to 2008. ^c^n birth certificate 1996 to 2008; ^d^Limited to participants with at least 1 recorded height/weight measurement between ages 0 to 24 months. ^e^Limited to participants with all 4 crossings between ages 0 to 24 months (e.g., 0–6, 6–12, 12–18, 18–24)

In Model 2, adjusting for child sex and gestational age at birth, pregnancies with missing paternal data had a 70 % reduced odds of breastfeeding initiation (AOR 0.30; 95 % CI: 0.29, 0.32) than pregnancies belonging to the group of married mothers with available paternal data. These pregnancies were also marked by lower birthweights (adjusted β -0.20 kg; 95 % CI: −0.21, −0.19) and an elevated odds of ever having a WFL ≥ 95th percentile (AOR 1.31; 95 % CI: 1.23, 1.40) and crossing ≥2 WFL percentiles before 24 months of age (AOR 1.19; 95 % CI: 1.01, 1.41). Among pregnancies without paternal data, adding maternal measures to the model (Model 3), resulted in a notable increase in the odds ratio of smoking during pregnancy (AOR 9.27; 95 % CI: 8.45, 10.17) and a significant association for the odds of gestational diabetes (AOR 0.78; 95 % CI: 0.62, 0.98). Adjustment for smoking during pregnancy in Model 4 slightly lowered the likelihoods of breastfeeding initiation (AOR 0.29; 95 % CI: 0.27, 0.31) and gestational diabetes (AOR 0.74; 95 % CI: 0.59, 0.93) and reduced infant birthweight (adjusted β -0.09 kg; 95 % CI: −0.10, −0.08). The point estimate for maternal smoking during pregnancy was attenuated after adjustment for maternal education in Model 5 (from 9.27 to 6.10; 95 % CI: 5.55, 6.72) and insurance status in Model 6 (from 6.10 to 4.40; 95 % CI: 3.97, 4.87). Adjustment for these factors also resulted in a significant association for the odds of children ever having a WFL ≥ 95th percentile before 24 months of age (AOR 1.10; 95 % CI: 1.01, 1.20).

Associations for the group of pregnancies with available paternal data involving unmarried mothers were similar to the group with missing paternal data, but the differences were less pronounced. After adjustment for child and maternal factors (Model 3), these pregnancies had increased odds of smoking during pregnancy (AOR 7.63; 95 %: CI: 7.19, 8.10), lower birthweights (adjusted β -0.07 kg; 95 % CI: −0.08, −0.06), and decreased odds of gestational diabetes (AOR 0.86; 95 % CI: 0.76, 0.97) and breastfeeding initiation (AOR 0.36; 95 % CI: 0.34, 0.37) than pregnancies involving married mothers with paternal data available. The associations for gestational diabetes, breastfeeding initiation, and birthweight remained significant in the models additionally adjusting for smoking during pregnancy (Model 4) and were slightly attenuated after adjustment for maternal education (Model 5) and insurance status (Model 6).

## Discussion

In this large cohort study, the availability of paternal data on birth certificates in Massachusetts was independently related to perinatal risk factors for childhood obesity. In adjusted analyses, we observed higher rates of smoking during pregnancy, lower rates of breastfeeding initiation, and reduced infant birthweight among pregnancies without paternal data on the birth certificate as compared to pregnancies involving married mothers where paternal data were available. We also observed higher odds of children ever having a WFL ≥ 95th percentile and of crossing ≥ 2 major WFL percentiles in the first 2 years of life, although this association was modestly attenuated after adjusting for maternal characteristics. Our results raise the possibility of using missing paternal data on the infant birth certificate as a practical tool to identify children who may be at greater risk for certain perinatal precursors of childhood obesity and suggest that efforts to understand and reduce childhood obesity risk factors in early life may need to consider paternal factors.

Our findings are consistent with published reports that relate missing paternal data on the birth certificate with obstetric, pregnancy, and early neonatal outcomes [14, 16, 20–23] and extend this knowledge base, for the first time, to the field of childhood obesity. Similar to a previous study in Georgia, we found that prevalence of adverse infant outcomes were higher among pregnancies missing paternal data than among pregnancies providing paternal data, whether women were married or not [23]. We are not aware of any studies that have examined outcomes after the pregnancy period except for infant mortality. Moreover, most prior studies of paternal effects on maternal health behaviors (e.g., prenatal care and smoking during pregnancy) have focused only on known fathers of primarily married couples [15]. Our dataset provided the opportunity to expand this work to families with missing paternal data, and to look at risk factors for obesity early in the life course. We observed higher rates of smoking during pregnancy and lower rates of breastfeeding initiation among pregnancies with missing paternal data. Paradoxically, pregnancies with missing paternal data had decreased odds of gestational diabetes. However the unadjusted rates of gestational diabetes were similar across the three study groups so it is possible that this finding was impacted by residual confounding by unmeasured variables such as maternal body mass index [27]. Finally, we observed a trend towards increased odds of early upwards crossing of major weight-for-length percentiles, an indicator of subsequent obesity risk [26], in the group with missing paternal data, but this effect was attenuated after adjustment for maternal age, race/ethnicity, and parity.

Consistent with a national study of twin births by Tan et al., we found pregnancies without paternal data tended to occur among mothers who were younger and of black race [20], characteristics that were distinct from unmarried mothers who provided paternal data, who tended to be comparatively older, nulliparous, and of white race. Though the risks for adverse infant outcomes were elevated for the group of unmarried mothers who provided paternal data, the differences were less pronounced than cases where paternal data were absent.

Previous studies have found associations between family structure and various aspects of child development, including risk for overweight/obesity [19, 28, 29]. In general, that work finds that children with divorced parents have a higher prevalence of poor health outcomes than children residing in single-parent households, who in turn have poorer health than children living with their biological, married parents [19, 29]. Obese children more frequently live in households with an unmarried single parent than non-obese children [19]. This is broadly consistent with our data. However, we also found that when compared to pregnancies involving married mothers, pregnancies missing paternal data had worse prenatal and infant outcomes related to childhood obesity than pregnancies involving unmarried mothers where paternal data were available. This suggests that marital status alone may be insufficient as an indicator of paternal risk. Vast changes to family systems have occurred in the US over recent decades, including substantial increases in cohabitation, non-marital childbearing, and single-parent families [30]. Today over 40 % of births in the US are to unmarried women [31], a group that encompasses both partnered and un-partnered women. Further, nearly 39 % of unmarried, cohabitating heterosexual couples report having at least one biological child of either partner residing in the household and approximately 50 % of unmarried women who give birth are cohabiting with the fathers of their children [32]. Together with our data, these facts underscore the importance of incorporating various family structures into investigations of the role of fathers to maternal prenatal, neonatal, and early child health.

We did not have information about why paternal variables were not reported on the birth certificate, but given the potential legal and financial repercussions of omitting paternal data (e.g., establishment of paternity, custodial and visitation rights, and the ability to collect child support) [33], one can assume that the absence of these data reflects some degree of emotional, physical and/or financial separation. Moreover, previous work has found that the proportion of unmarried fathers with information on the birth certificate (90 %) is similar to the proportion who were involved during the pregnancy (87 %) and planned to be involved during the child’s life (91 %) [16], providing support for this interpretation. Future research should explore the reasons for missing paternal data on birth certificates and investigate the association of missing paternal data with other aspects of maternal and infant health. To this end, qualitative research may be particularly important to help researchers identify factors associated with missing birth certificate data, which could reveal specific types family structures that may be more at risk for adverse health outcomes.

This study has several limitations. First, although vital records are an important resource for identifying factors associated with suboptimal perinatal outcomes, birth certificate data may incorrectly report some information. For example, maternal smoking during pregnancy is under-reported on the birth certificate compared to data on smoking collected on confidential surveys completed postpartum [34]. The birth certificate also under-reports gestational diabetes [35]. The birth certificate did not collect data on maternal smoking during pregnancy and gestational diabetes throughout the duration of the study period. We were unable to match all CENTURY cohort cases to our linked dataset. Children not linked were more likely than children in our study to be born in the 1970s and 1980s, be from an ethnic minority group, or have missing race/ethnicity or medical insurance information [24]. This may have biased our findings in an unknown direction. Our findings are also specific to a cohort of children residing in Massachusetts and may not be generalizable to other populations. Finally, our results may be confounded by important unmeasured variables, such as household income, that are plausibly related to father involvement and our outcomes of interest. Maternal body mass index is also strong predictor of childhood obesity [36] but was not assessed on the Massachusetts birth certificate during the period of investigation.

## Conclusions

Obesity rates among children have substantially increased worldwide over the past 3 decades [37]. The family context presents an opportunity for early intervention and prevention, but most studies of family influences to children’s obesity outcomes have focused on maternal factors including gestational weight gain [38], smoking during pregnancy [39], overweight status [40], psychosocial stress and depression [41], and control of infant feeding [42, 43]. The influence of fathers is largely missing from these efforts. Yet paternal engagement (e.g., involvement in pregnancy-related activities and infant feeding practices), responsibility (e.g., commitment to ensuring the child’s care and welfare, including financial support), and quality of the relationship with mothers are likely key influences in shaping the family environment that leads to the development of child obesity. Our findings lend support for further efforts to understand the childhood obesity epidemic within the context of children’s socio-environmental and familial contexts and draw attention to the potential unique contribution of fathers. More research is needed to identify mechanisms by which the absence of paternal data is associated with an increased burden of poor pregnancy and infant health outcomes and into the biological and social pathways by which fathers influence children’s obesity rates, which could refine prevention and intervention efforts.

## Ethics approval and consent to participate

Institutional Review Board approval for the CENTURY Study was obtained from Boston College, Harvard Pilgrim Health Care (HPHC), MDPH, and the Massachusetts General Hospital for Children.

## Consent for publication

Not applicable.

## Availability of data and materials

Data will not be shared. These data have been obtained from the Department of Public Health. As recipients, the authors were permitted to publish any analyses of the data, but not the data itself, due to confidentiality conditions.

## Appendix

**Table 3:** Unadjusted and Multivariable Adjusted Associations* of 3-category Exposure with Perinatal Risk Factors for Childhood Obesity, data from the Linked Century Study 2004–2008 (*N* = 30,014)

		Model 1	Model 2	Model 3	Model 4	Model 5	Model 6
Outcome	Exposure	OR (95 % CI)					
Smoked during pregnancy							
	Missing paternal data	11.08 (9.03,13.60)	11.09 (9.03,13.61)	19.06 (15.25,23.81)	-	13.39 (10.65,16.84)	7.37 (5.75, 9.44)
	Paternal data, not married	10.26 (8.92,11.79)	10.26 (8.93,11.80)	13.55 (11.67,15.74)	-	10.33 (8.85,12.04)	6.09 (5.12, 7.25)
	Paternal data, married	1.00 (Reference)	1.00 (Reference)	1.00 (Reference)	-	1.00 (Reference)	1.00 (Reference)
Gestational diabetes							
	Missing paternal data	0.99 (0.73, 1.35)	0.99 (0.73, 1.35)	0.98 (0.71, 1.35)	0.95 (0.69, 1.30)	0.98 (0.71, 1.35)	1.00 (0.71, 1.39)
	Paternal data, not married	0.82 (0.69, 0.98)	0.82 (0.69, 0.98)	0.88 (0.73, 1.06)	0.85 (0.70, 1.03)	0.87 (0.72, 1.06)	0.89 (0.72, 1.08)
	Paternal data, married	1.00 (Reference)	1.00 (Reference)	1.00 (Reference)	1.00 (Reference)	1.00 (Reference)	1.00 (Reference)
Initiated breastfeeding							
	Missing paternal data	0.32 (0.28, 0.37)	0.33 (0.29, 0.38)	0.21 (0.18, 0.25)	0.27 (0.23, 0.32)	0.31 (0.26, 0.36)	0.36 (0.30, 0.43)
	Paternal data, not married	0.36 (0.33, 0.39)	0.36 (0.33, 0.39)	0.27 (0.25, 0.30)	0.34 (0.31, 0.37)	0.37 (0.34, 0.41)	0.42 (0.38, 0.47)
	Paternal data, married	1.00 (Reference)	1.00 (Reference)	1.00 (Reference)	1.00 (Reference)	1.00 (Reference)	1.00 (Reference)
WFL ever ≥95th percentile, 0–24 mo^a^							
	Missing paternal data	1.39 (1.20, 1.63)	1.38 (1.18, 1.61)	1.21 (1.03, 1.42)	1.16 (0.99, 1.37)	1.18 (1.00, 1.39)	1.17 (0.99, 1.39)
	Paternal data, not married	1.28 (1.17, 1.39)	1.29 (1.18, 1.40)	1.19 (1.09, 1.31)	1.16 (1.05, 1.27)	1.17 (1.06, 1.29)	1.16 (1.05, 1.29)
	Paternal data, married	1.00 (Reference)	1.00 (Reference)	1.00 (Reference)	1.00 (Reference)	1.00 (Reference)	1.00 (Reference)
Crossed ≥2 major WFL percentiles, 0–24 mo^b^							
	Missing paternal data	1.47 (0.93, 2.33)	1.46 (0.93, 2.31)	1.45 (0.91, 2.31)	1.43 (0.90, 2.28)	1.50 (0.94, 2.39)	1.52 (0.94, 2.45)
	Paternal data, not married	0.91 (0.74, 1.12)	0.92 (0.75, 1.14)	0.92 (0.74, 1.15)	0.90 (0.72, 1.12)	0.94 (0.75, 1.17)	0.95 (0.75, 1.20)
	Paternal data, married	1.00 (Reference)	1.00 (Reference)	1.00 (Reference)	1.00 (Reference)	1.00 (Reference)	1.00 (Reference)
		β (95 % CI)					
Birthweight, kg							
	Missing paternal data	−0.20 (−0.24,-0.17)	−0.18 (−0.21,-0.15)	−0.13 (−0.16,-0.10)	−0.10 (−0.13,-0.07)	−0.09 (−0.12,-0.06)	−0.07 (−0.10,-0.04)
	Paternal data, not married	−0.11 (−0.13,-0.09)	−0.11 (−0.13,-0.10)	−0.08 (−0.09,-0.06)	−0.05 (−0.07,-0.03)	−0.05 (−0.06,-0.03)	−0.03 (−0.05,-0.01)
	Paternal data, married	0.00 (Reference)	0.00 (Reference)	0.00 (Reference)	0.00 (Reference)	0.00 (Reference)	0.00 (Reference)

**WFL* weight-for-length, *kg* kilograms, *OR*, odds ratio, *CI* confidence interval. Model 1 is unadjusted. Model 2 adjusted for child gestational age at birth and sex. Model 3 added maternal age (≥35 years), parity (nulliparous), and race/ethnicity. Model 4 added smoking during pregnancy. Model 5 added maternal education. Model 6 added insurance status. Smoking during pregnancy and gestational diabetes models were not adjusted for gestational age at birth

^a^Limited to 27,073 participants with at least 1 recorded height/weight measurement between ages 0 to 24 months. ^b^Limited to 4,190 participants with all 4 crossings between ages 0 to 24 months (e.g., 0–6, 6–12, 12–18, 18–24)

## Abbreviations

CI: confidence interval
HPHC: Harvard pilgrim health care
HVMA: Harvard vanguard medical associates
MDPH: Massachusetts department of public health
OR: odds ratio
WFL: weight for length

## References

[1] General Assembly of the United Nations: High-level Meeting on Non-communicable Diseases. In. http://www.un.org/en/ga/president/65/issues/ncdiseases.shtml; Accessed June 1, 2011; 2011.

[2] Martin JA, Hamilton BE, Osterman MJ, Curtin SC, Matthews TJ (2015). Births: final data for 2013. Natl Vital Stat Rep.

[3] Ogden CL, Carroll MD, Kit BK, Flegal KM (2014). Prevalence of childhood and adult obesity in the United States, 2011–2012. JAMA.

[4] Franks PW, Hanson RL, Knowler WC, Sievers ML, Bennett PH, Looker HC (2010). Childhood obesity, other cardiovascular risk factors, and premature death. N Engl J Med.

[5] Biro FM, Wien M (2010). Childhood obesity and adult morbidities. Am J Clin Nutr.

[6] Sinha R, Fisch G, Teague B, Tamborlane WV, Banyas B, Allen K, Savoye M, Rieger V, Taksali S, Barbetta G (2002). Prevalence of impaired glucose tolerance among children and adolescents with marked obesity. N Engl J Med.

[7] McCance RA, Widdowson EM (1974). The determinants of growth and form. Proc R Soc Lond B Biol Sci.

[8] Plagemann A, Heidrich I, Gotz F, Rohde W, Dorner G (1992). Obesity and enhanced diabetes and cardiovascular risk in adult rats due to early postnatal overfeeding. Exp Clin Endocrinol.

[9] World Health Organization (2008). Commission on the Social Determinants of Health.

[10] Dixon B, Pena MM, Taveras EM (2012). Lifecourse approach to racial/ethnic disparities in childhood obesity. Adv Nutr.

[11] Freeman E, Fletcher R, Collins CE, Morgan PJ, Burrows T, Callister R (2012). Preventing and treating childhood obesity: time to target fathers. Int J Obes (Lond).

[12] Alio AP, Kornosky JL, Mbah AK, Marty PJ, Salihu HM (2010). The impact of paternal involvement on feto-infant morbidity among Whites, Blacks and Hispanics. Matern Child Health J.

[13] Alio AP, Bond MJ, Padilla YC, Heidelbaugh JJ, Lu M, Parker WJ (2011). Addressing policy barriers to paternal involvement during pregnancy. Matern Child Health J.

[14] Alio AP, Mbah AK, Kornosky JL, Wathington D, Marty PJ, Salihu HM (2011). Assessing the impact of paternal involvement on racial/ethnic disparities in infant mortality rates. J Community Health.

[15] Martin LT, McNamara MJ, Milot AS, Halle T, Hair EC (2007). The effects of father involvement during pregnancy on receipt of prenatal care and maternal smoking. Matern Child Health J.

[16] Teitler JO (2001). Father involvement, child health and maternal health behavior. Child Youth Serv Rev.

[17] Khandpur N, Blaine RE, Fisher JO, Davison KK (2014). Fathers’ child feeding practices: A review of the evidence. Appetite.

[18] Hohwu L, Zhu JL, Graversen L, Li J, Sorensen TI, Obel C (2015). Prenatal parental separation and body weight, including development of overweight and obesity later in childhood. PLoS One.

[19] Gable S, Lutz S (2000). Household, Parent, and Child Contributions to Childhood Obesity. Fam Relat.

[20] Tan H, Wen SW, Walker M, Demissie K (2004). Missing paternal demographics: A novel indicator for identifying high risk population of adverse pregnancy outcomes. BMC Pregnancy Childbirth.

[21] Getahun D, Ananth CV, Vintzileos AM (2006). Uteroplacental bleeding disorders during pregnancy: do missing paternal characteristics influence risk?. BMC Pregnancy Childbirth.

[22] Fulda KG, Kurian AK, Balyakina E, Moerbe MM (2014). Paternal race/ethnicity and very low birth weight. BMC Pregnancy Childbirth.

[23] Gaudino JA, Jenkins B, Rochat RW (1999). No fathers’ names: a risk factor for infant mortality in the State of Georgia. USA Soc Sci Med.

[24] Hawkins SS, Gillman MW, Rifas-Shiman SL, Kleinman KP, Mariotti M, Taveras EM (2016). The Linked CENTURY Study: linking three decades of clinical and public health data to examine disparities in childhood obesity. BMC Pediatr.

[25] Kim J, Peterson KE, Scanlon KS, Fitzmaurice GM, Must A, Oken E, Rifas-Shiman SL, Rich-Edwards JW, Gillman MW (2006). Trends in overweight from 1980 through 2001 among preschool-aged children enrolled in a health maintenance organization. Obesity (Silver Spring).

[26] Taveras EM, Rifas-Shiman SL, Sherry B, Oken E, Haines J, Kleinman K, Rich-Edwards JW, Gillman MW (2011). Crossing growth percentiles in infancy and risk of obesity in childhood. Arch Pediatr Adolesc Med.

[27] Torloni MR, Betran AP, Horta BL, Nakamura MU, Atallah AN, Moron AF, Valente O (2009). Prepregnancy BMI and the risk of gestational diabetes: a systematic review of the literature with meta-analysis. Obes Rev.

[28] Augustine A, Kimbro RT (2013). Family structure and obesity among US children. J Appl Res Child.

[29] Waldfogel J, Craigie TA, Brooks-Gunn J (2010). Fragile families and child wellbeing. Future Child.

[30] Cherlin AJ (2005). American marriage in the early twenty-first century. Future Child.

[31] Martin JA, Hamilton BE, Osterman MJ (2013). Births in the United States. NCHS Data Brief.

[32] Kennedy S, Bumpass L (2008). Cohabitation and children’s living arrangements: New estimates from the United States. Demogr Res.

[33] Abrams DE, Ramsey SH (2007). Children and the law: Doctrine, policy, and practice.

[34] Allen AM, Dietz PM, Tong VT, England L, Prince CB (2008). Prenatal smoking prevalence ascertained from two population-based data sources: birth certificates and PRAMS questionnaires, 2004. Public Health Rep.

[35] Devlin HM, Desai J, Walaszek A (2009). Reviewing performance of birth certificate and hospital discharge data to identify births complicated by maternal diabetes. Matern Child Health J.

[36] Gillman MW, Rifas-Shiman SL, Kleinman K, Oken E, Rich-Edwards JW, Taveras EM (2008). Developmental origins of childhood overweight: potential public health impact. Obesity (Silver Spring).

[37] Wang Y, Lobstein T (2006). Worldwide trends in childhood overweight and obesity. Int J Pediatr Obes.

[38] Oken E, Rifas-Shiman SL, Field AE, Frazier AL, Gillman MW (2008). Maternal gestational weight gain and offspring weight in adolescence. Obstet Gynecol.

[39] Oken E, Levitan EB, Gillman MW (2008). Maternal smoking during pregnancy and child overweight: systematic review and meta-analysis. Int J Obes (Lond).

[40] Gibson LY, Byrne SM, Davis EA, Blair E, Jacoby P, Zubrick SR (2007). The role of family and maternal factors in childhood obesity. Med J Aust.

[41] Gundersen C, Mahatmya D, Garasky S, Lohman B (2011). Linking psychosocial stressors and childhood obesity. Obes Rev.

[42] Harder T, Bergmann R, Kallischnigg G, Plagemann A (2005). Duration of breastfeeding and risk of overweight: a meta-analysis. Am J Epidemiol.

[43] Baker JL, Michaelsen KF, Rasmussen KM, Sorensen TI (2004). Maternal prepregnant body mass index, duration of breastfeeding, and timing of complementary food introduction are associated with infant weight gain. Am J Clin Nutr.

